# RPL15 promotes hepatocellular carcinoma progression via regulation of RPs-MDM2-p53 signaling pathway

**DOI:** 10.1186/s12935-022-02555-5

**Published:** 2022-04-11

**Authors:** Rui Shi, Zirong Liu

**Affiliations:** Department of Hepatobiliary Surgery, Tianjin First Central Hospital, No. 24, Fukang Rd., Tianjin, 300192 Nankai District China

**Keywords:** Hepatocellular carcinoma, MDM2, p53, RPL15, Tumor progression

## Abstract

**Backround:**

RPL15 has been found to participate in human tumorigenesis. However, its function and regulatory mechanism in hepatocellular carcinoma (HCC) development are still unclear. Current study investigated the effects of RPL15 in HCC.

**Methods:**

The expression of RPL15 in clinical tissues and cell lines of HCC was detected by RT-qPCR, Western blotting, and Immunohistochemistry (IHC). Colony formation, CCK-8, flow cytometry, Wound healing and Transwell invasion assays, were used to detect the carcinoma progression of HCC cells with RPL15 overexpression or knockdown in vitro. A xenograft model was constructed to assess the effect of RPL15 knockdown on HCC cells in vivo. The expression of CDK2 and Cyclin E1 related to cell cycles, Bax and Bcl-2 related to cell apoptosis, E-cadherin, N-cadherin and Vimentin related to epithelial–mesenchymal transition (EMT), p53 and p21 related to p53 signaling pathway, were detected by Western blotting. The connection between p53, MDM2 and RPL5/11 affected by RPL15 was analyzed using immunoprecipitation and Cycloheximide (CHX) chase assay.

**Results:**

Elevated RPL15 was identified in HCC tissues, which was not only a prediction for the poor prognosis of HCC patients, but also associated with the malignant progression of HCC. RPL15 silencing arrested HCC cell cycle, suppressed HCC cell colony formation, proliferation, invasion, and migration, and induce cell apoptosis. On the contrary, RPL15 upregulation exerted opposite effects. Results also indicated that HCC cell invasion and migration were associated with EMT, and that the RPs-MDM2-p53 pathway was implicated in RPL15-mediated oncogenic transformation. In addition, RPL15 knockdown significantly suppressed HCC xenografts growth.

**Conclusions:**

RPL15 played crucial roles in HCC progression and metastasis, serving as a promising candidate for targeted therapies.

**Supplementary Information:**

The online version contains supplementary material available at 10.1186/s12935-022-02555-5.

## Introduction

Hepatocellular carcinoma (HCC) is one of the malignant tumors that seriously threaten human health. According to the cancer statistics from the World Health Organization, there are approximately 905,677 new cases of HCC and 830,180 deaths from HCC worldwide in 2020, and the mortality rate of HCC is as high as 8.3%, ranking third in the world [1]. Clinically, the main treatments for HCC are surgical resection, liver transplantation, and local ablation. However, most patients with HCC have hidden onset and are already at an advanced stage or have metastases at the time of diagnosis, losing the opportunity for radical treatment [2]. In addition, due to the rapid development, the poor prognosis and the extremely high mortality rate, the curative effect of HCC is still not ideal, and it is easy to cause drug resistance, recurrence, and low survival rate even if some radiotherapy or chemotherapy methods are adopted [3]. Therefore, in-depth study of the molecular mechanism of HCC occurrence and development can lay a theoretical foundation for early diagnosis and specific treatment.

The ribosomes are composed of RNA and protein, and are the site of protein synthesis in cells. By controlling ribosome formation and protein synthesis, ribosome biogenesis plays a vital role in cell growth, stress response, and the transmission of genetic information [4]. Abnormality of ribosomal biogenesis will lead to abnormal protein synthesis in cells, cause disturbances in cell life activities, and can lead to many human diseases, such as anemia and tumors [5, 6]. For example, congenital aplastic anemia (DBA) or 5q syndrome is mainly caused by the deletion or mutation of ribosomal large and small subunit proteins such as RPL11 or RPS14, resulting in abnormal assembly and maturation of ribosomal large and small subunits, ultimately leading to macrocytic anemia, growth retardation, and acute myeloid leukemia [7–9]. The occurrence of a variety of tumors is closely related to the mutation and abnormal expression of ribosomal-related proteins. Except for the overexpression of a variety of large-subunit ribosomal proteins (RPLs) and small-subunit ribosomal proteins (RPSs) in human tumors, RPL11, RPL5 and RPL10 have detected mutations in T cell-acute lymphocytic leukemia, and RPS20 and RPL15 has detected mutations in colon cancer [10, 11].

RPL15 (ribosomal protein L15) is a component of the ribosomal large subunit 60S, participating in the assembly process of ribosomal subunits and involving in the processing of rRNA [12]. Moreover, RPL15 deletion will lead to abnormalities in the biogenesis of ribosomal subunits [13]. The abnormal expression or mutation of RPL15 protein is also closely related to the occurrence and development of many diseases. Studies by Landowski M showed that the gene deletion of RPL15 was identified in congenital aplastic anemia [14]. Moreover, the expression of RPL15 varies in different tumor cancer cells. For example, the high expression of RPL15 in esophageal cancer was involved in the carcinogenesis [15]. RPL15 was highly expressed in gastric cancer cell lines, and interference with the expression of RPL15 could inhibit gastric cancer cell growth [16]. In addition, RPL15 in skin squamous cell carcinoma tissues and pancreatic cancer cell lines decreased sharply, which had a great relationship with the survival rate [17, 18]. To date, little is known about the role of RPL15 in HCC.

In this study, we investigated the role of RPL15 in Hepatocellular carcinoma (HCC). Our study found that RPL15 was overexpressed in HCC cells and tissues, and the expression of RPL15 was closely associated with hepatocellular carcinome. Furthermore, we demonstrated that RPL15 silencing induced HCC cell apoptosis, and inhibited the proliferation, colony formation, migration, and invasion of HCC cells. These results supported the potential value of RPL15 as a therapeutic target in HCC treatment.

## Materials and methods

### Tissue samples

HCC and para-carcinoma tissues were obtained from 15 HCC patients who underwent curative surgery at Tianjin First Central Hospital. No patient had received chemotherapy or radiotherapy prior to surgery. The morphology was verified independently in a double-blind manner by at least two expert pathologists. This study was approved by the Ethics Committee of Tianjin First Central Hospital (E2020011L), and all patients provided the written informed consent.

### Cell lines and cell culture

An immortalized normal liver cell line (LO2) and several HCC cell lines (Huh7, HCCLM3, Hep3B and HepG2) were purchased from the Institute of Biochemistry and Cell Biology (Chinese Academy of Sciences, Shanghai, China).

Cells harbor wild-type p53 (HepG2, HCCLM3 and LO2), non-sense p53 mutation (Hep3B) and p53 point mutation (Huh7). All cell lines were cultured in DMEM medium which was supplemented with 10% fetal bovine serum (10099141C, Thermo Scientific, MA, USA) at 37 °C in a 5% CO_2_ incubator (Thermo Scientific, MA, USA).

### Cell transfection

The mixture of two siRNAs specific targeting to RPL15 (1#: 5'-UGGUGUUAACCAGCUAAAGdTdT-3'; 2#: 5'-UCCAGGAGCUAUGGAGAAAdTdT-3') and scramble siRNA as the negative control were synthesized by Gene pharma (Shanghai, China) [11]. The cDNA encoding RPL15 was inserted into the pcDNA-3.1 plasmid (V79020, Thermo Scientific, MA, USA), and the empty vector was served as negative control. The above siRNAs and vectors were transfected into HCC cells by Lipofectamine 3000 reagent (L3000015, Thermo Scientific, MA, USA). The transfection efficiency was the higher when the plasmid dosage was 2 ug and the siRNA was 100 pmol for 35 mm dish.

### qRT-PCR

Total RNA was extracted using TRIzol reagent (15596026, Thermo Scientific, MA, USA). Reverse transcription was performed to synthesize cDNA using a PrimeScript RT Master Mix Perfect Real-Time kit (RR036A, Takara Bio Inc, Liaoning, China.). RT-qPCR was performed using SYBR Green PCR Master mix (RR420A, Takara Bio Inc, Liaoning, China) and StepOnePlus Real-Time PCR System (Thermo Scientific, MA, USA). The relative expression of mRNAs was quantified using the 2^−∆∆Ct^ method with GAPDH as an internal control. Primers for real-time PCR used were as follows: RPL15:5′- ACAAGGCCAAGCAAGGTTAC-3′, 5′- ACTGAAGGCTTCGAGCAAAC-3′, GAPDH: 5′-GCATTGCCCTCAACGACCAC-3′, 5′-CCACCACCCTGTTGCTGTAG-3′, and synthesized by Gene pharma (Shanghai, China).

### CCK-8 assay

Cell viability was detected at 48 h using a CCK-8 kit (CK04, Dojindo, Tokyo, Japan). The absorbance at 450 nm was measured using a microplate reader (Bio-Rad, CA, USA).

### Colony formation assay

Cells were plated in 6-well plates (500 per/well). The medium was replaced every 3 or 4 days. When cell colonies were formed after 14 days, the colonies were clearly visible and countable, and then they were fixed and stained with crystal violet. First, add 1 mL methanol to each well for 5 min, discarding the methanol and drying at room temperature. Crystal violet (1 mL) (C0121, Biyotime, Shanghai, China) was added to each well and allowed to stain for 5 min. Then washed with ultrapure water and immersed in 1 mL acetic acid (33%) to dissolve the stain, repeat washing 3 times. Images were captured with Olympus BX51 microscope (Olympus Corporation, Tokyo, Japan) at 4× magnification.

### Wound healing assay

Wound healing assay was performed to detect HCC cells migration. Cells were seeded in 24-well plates. A scratch wound was generated by a pipette tip. Afterwards, the cells were cultured for another 24 h. Photographs were taken after 0 h and 24 h under an Olympus BX51 microscope (Olympus Corporation, Tokyo, Japan) at 4× magnification.

### Transwell invasion assay

Transwell chambers (3422, Corning, NY, USA) pre-coated with Matrigel (356234, BD Biosciences, NJ, USA) were used to measure cell invasion ability. HCC cells were suspended in serum-free medium and added to the upper chambers and incubated at 37 °C for 24 h. DMEM medium containing 10% FBS was placed in the lower chambers. The invasion cells were fixed and stained, then 5 fields per chamber (insert) were observed for counting under an Olympus BX51 microscope (Olympus Corporation, Tokyo, Japan) at 10× magnification.

### Western blot

Total protein was extracted from cells transfected 48 h with RIPA lysis buffer (P0013C, Biyotime, Shanghai, China) containing protease inhibitors. The protein concentration of the lysates was analyzed by BCA protein assay kit (23227, Thermo Scientific, MA, USA). 40 μg of protein was separated on a 10% SDS-polyacrylamide gel and blotted onto polyvinylidene difluoride (PVDF) membranes (IPVH00010, Merck-Millipore, NJ, USA). After blocking with 5% skim milk for 1 h, the membranes were then incubated with primary antibodies, RPL15 (1:1000; Abcam, ab155802, MA, USA), CDK2 (1:2000; Abcam, ab32147, MA, USA), Cyclin E1 (1:1000; Abcam, ab224819, MA, USA), Bcl-2 (1:1000; Abcam, ab218123, MA, USA), Bax (1:2000; Abcam, ab3191, MA, USA), E-cadherin (1:20,000; Abcam, ab49772, MA, USA), N-cadherin (1:5000; Abcam, ab98952, MA, USA), vimentin (1:2000; Abcam, ab271673, MA, USA), p21 (1:3000; Abcam, ab188224, MA, USA), p53 (1:1000; Abcam, ab32049, MA, USA), MDM2 (1:1000; Abcam, ab16895, MA, USA), RPL5 (1 ug/ml; Abcam, ab86863, MA, USA), RPL11 (1:3000; Abcam, ab79352, MA, USA) overnight at 4 °C and HRP-conjugated secondary antibody (1:5000; 111-035-144, Jackson ImmunoResearch Inc., PA, USA) for 1 h at room temperature, β-actin (1:3000; Abcam, ab179467, MA, USA) was performed as an internal control. Immunoreactive signals were detected using the ECL detection system (WBKLS0100, Merck-Millipore, NJ, USA).

### Immunohistochemistry

The tissue specimens were fixed with 4% paraformaldehyde, dehydrated with graded ethanol, embedded in paraffin, and cut into 4-μm-thick paraffin sections. After that, the tissue section was deparaffinized in xylene and then rehydrated with gradient alcohol. Antigen retrieval was carried out in citrate buffer by microwaving the sections. Endogenous peroxidase activities were eliminated with 3% H_2_O_2_. Then, the sections were incubated with primary RPL15 (1:200; Abcam, ab155802, MA, USA) overnight at 4 °C, followed by being incubated with SABC-HRP Kit (P0615, Beyotime Biotechnology, Shanghai, China). The sections were then treated with DAB, and hematoxylin was used as the counterstain. The expression status of RPL15 in the nucleus was determined with the average percentage of positive cells in 5 random fields using a light microscope (Olympus BX51; Olympus Corporation, Tokyo, Japan) at 20× magnification.

### Cell cycle analysis

The transfected cells were fixed with cold 75% ethanol overnight at 4 °C. Thereafter, propidium iodide solution containing RNase were added and incubated in the dark at 37 °C for 30 min. Next, the cells were counted by flow cytometry on FACS C6 plus (BD Biosciences, CA, USA). Dates were analyzed using FlowJo software (BD Biosciences, CA, USA).

### Apoptosis analysis

HCC cells were resuspended in ice-cold PBS and Annexin V-fluorescein isothiocyanate (FITC)/PI staining was performed. Briefly, the cell suspension was incubated with Annexin V-FITC and PI for 30 min in the dark at room temperature; then the apoptotic cells were counted by flow cytometer.

### Immunoprecipitation

Cells were treated with IP lysis buffer (P0013, Biyotime, Shanghai, China), and followed by immunoprecipitation with the appropriate antibody at 4 °C overnight. Then, protein A/G plus-agarose (sc-2003, Santa Cruz, TX, USA) were added and incubated at 4 °C for 4 h. After five washes with lysis buffer, target antigens were cleared with loading buffer and prepared for western blotting analysis.

### Establish RPL15 stabl knockdown cell line

#### Xenograft model in nude mice

Animal protocols were approved by the Institutional Animal Care and Use Committee of Tianjin First Central Hospital Hospital. The BALB/c female nude mice (4 weeks old) were randomly grouped into two groups (n = 10 per group). LV3-shRPL15 or LV3 control plasmid was transfected into HCCLM3 cells by using Lentivirus packaging systems (GenePharma, Shanghai, China) to establish RPL15 stable knockdown cell line and negative control cell line. 2 $\times$ 10^6^ HCCLM3 cells with RPL15 stably depressed or negative control in 200 µl phosphate buffered saline, were injected subcutaneously to the right of the dorsal midline in nude mice. After the xenograft formed, the tumor size was evaluated with calipers every 3 days. On the 22nd day, we sacrificed the mice and removed the tumors for follow-up studies. The euthanasia method was followed as injecting sodium pentobarbital (200 mg/kg) into the left hip of mouse. The volume of the tumor was calculated using the following formula: tumor volume = 1/2 × length × width^2^.

### Statistical analysis

All experiments were repeated a minimum of 3 times. All statistical analyses were performed by GraphPad Prism 8.0 (GraphPad Software, CA, USA) or statistical software SPSS 18.0 (SPSS Inc., IL, USA). The student’s t test was used to analyze differences between two independent groups. One-way analysis of variance (ANOVA) was used for comparisons between multiple groups. Kaplan–Meier survival analysis and a Log rank test were used to evaluate the survival curve of the HCC patients. Results were considered statistically significant when P < 0.05.

## Results

### RPL15 was upregulated in human HCC tissues and cells

**Fig. 1:**
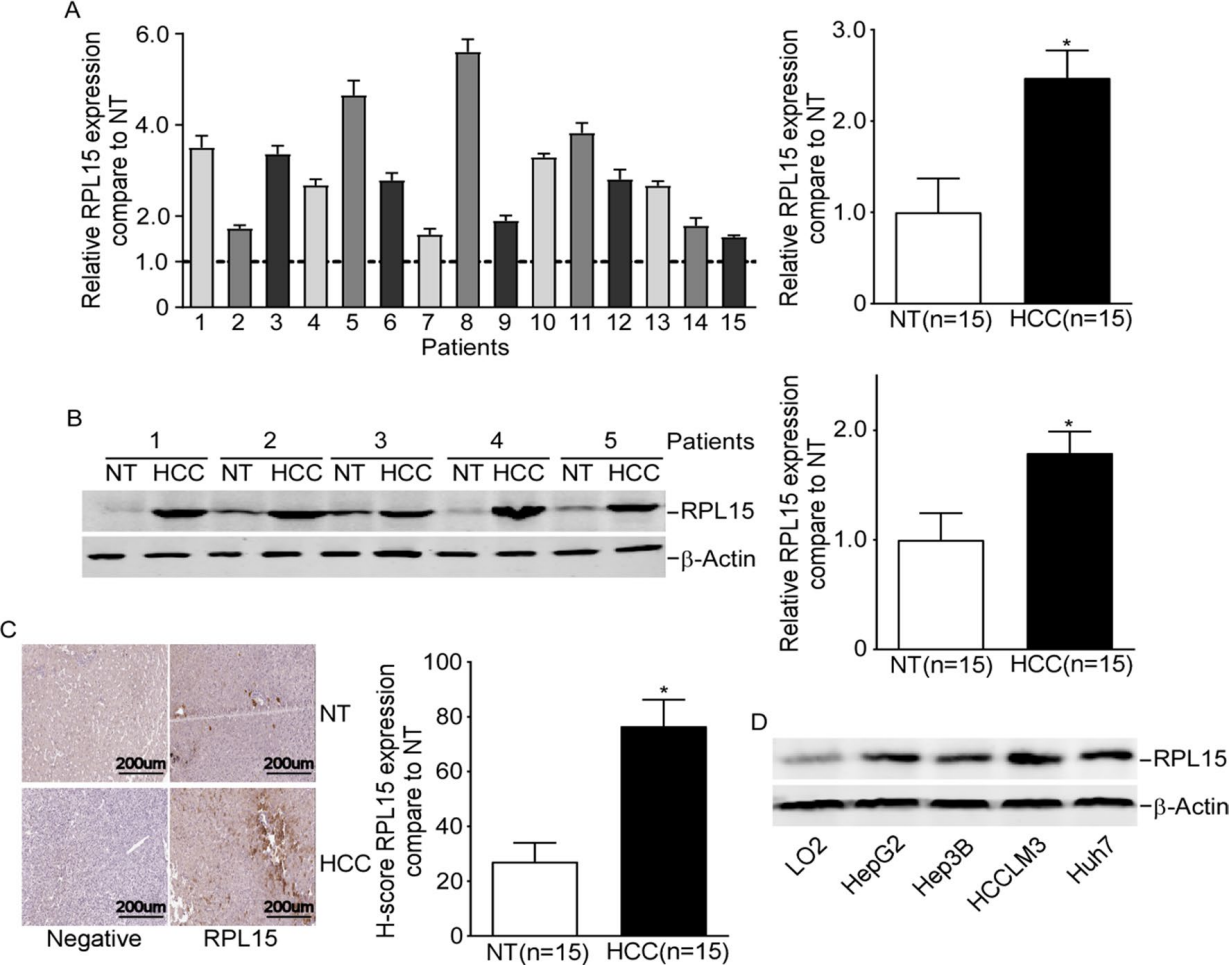
RPL15 was upregulated in human HCC tissues and cells. **A, B, C** Results of RT-qPCR, western blot and immunohistochemical analysis showed that RPL15 was upregulated in human HCC tissues. **D** Increased RPL15 levels were also detected in HCC cells. Data are presented as the mean ± SD, n = 3; *P < 0.05, compared to the control group

To confirm the biological functions of RPL15 in HCC, the RPL15 levels in HCC tissue samples and cells were detected. As demonstrated by RT-qPCR, elevated RPL15 was identified in HCC tissues (Fig. 1A). Moreover, the upregulation of RPL15 in HCC tissues was further confirmed by western blot (Fig. 1B). Immunohistochemical analysis showed strong nuclei staining of RPL15 (upregulated) in HCC tissues but little positive staining in normal tissues (Fig. 1C). Significantly increased RPL15 levels were also detected in the all HCC cells compared to the normal liver cell line by western blot (Fig. 1D).

### RPL15 overexpression predicted poor prognosis of HCC

**Fig. 2:**
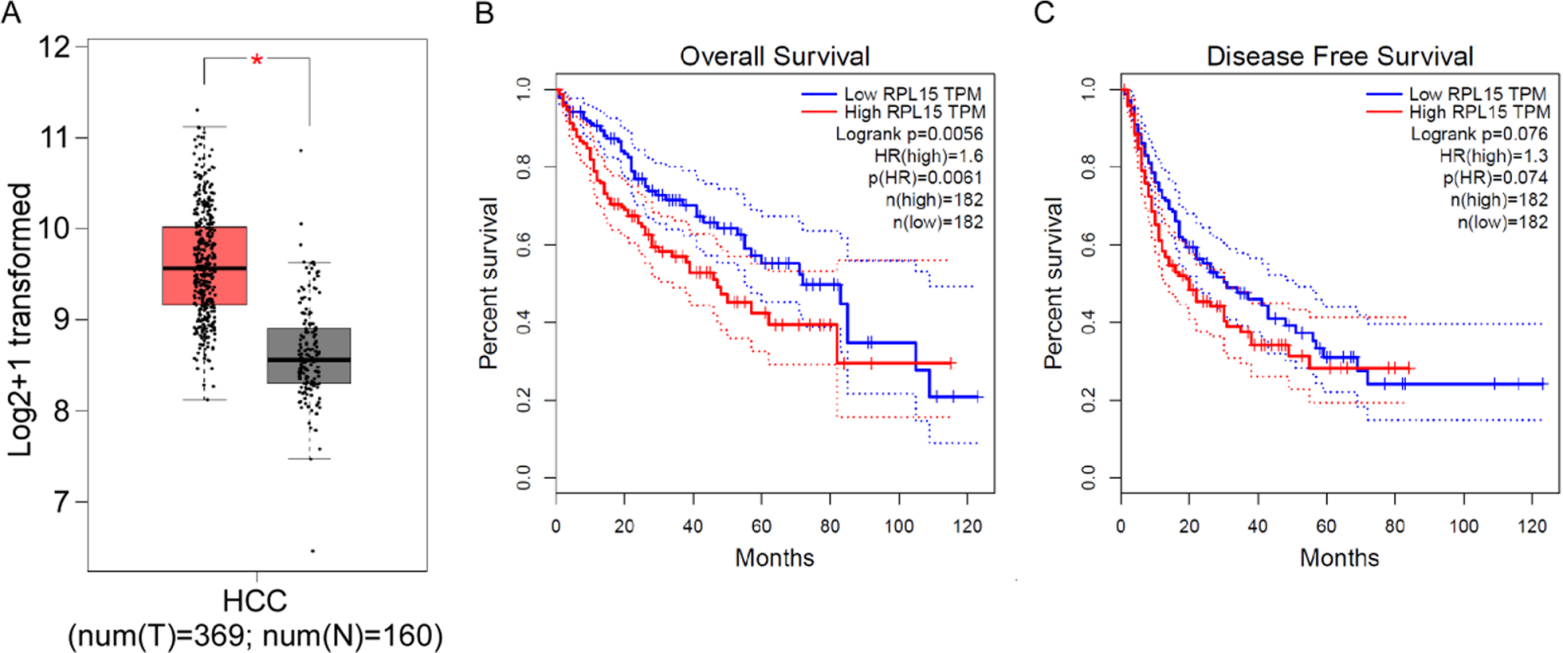
RPL15 unregulated in HCC predicted poor prognosis of HCC patients. **A** The expression of RPL15 was unregulated in T (tumor tissue) compared to N (negative para-carcinoma tissue) in TCGA HCC dataset**. B, C** Kaplan–Meier analysis indicated that HCC patients with high RPL15 expression displayed a significantly shorter overall survival (OS), but not obvious disease-free survival (DFS), the dotted lines mean 95% Confidengce Interval. *P < 0.05, compared to the control group

By analyzing the expression profile of RPL15 in HCC through compiling numerous open-access datasets from The Cancer Genome Atlas (TCGA), there was a significant increase of RPL15 transcription in HCCs (n = 369) compared to that in NTs (n = 160) (Fig. 2A). Additionally, the Kaplan–Meier analyses were used to evaluate the prognostic value of RPL15 in HCC patients. The results indicated that HCC patients in high RPL15 expression group displayed a significantly shorter overall survival (OS) rate than those in low RPL15 expression group, but no obvious difference with disease-free survival (DFS) rate (Fig. 2B, C). Taken together, these data suggested that upregulated RPL15 in HCC predicted poor prognosis of HCC patients and may contribute to malignant progression of HCC.

### Knockdown of RPL15 inhibited HCC cell proliferation and tumor growth both in vitro and in vivo

**Fig. 3:**
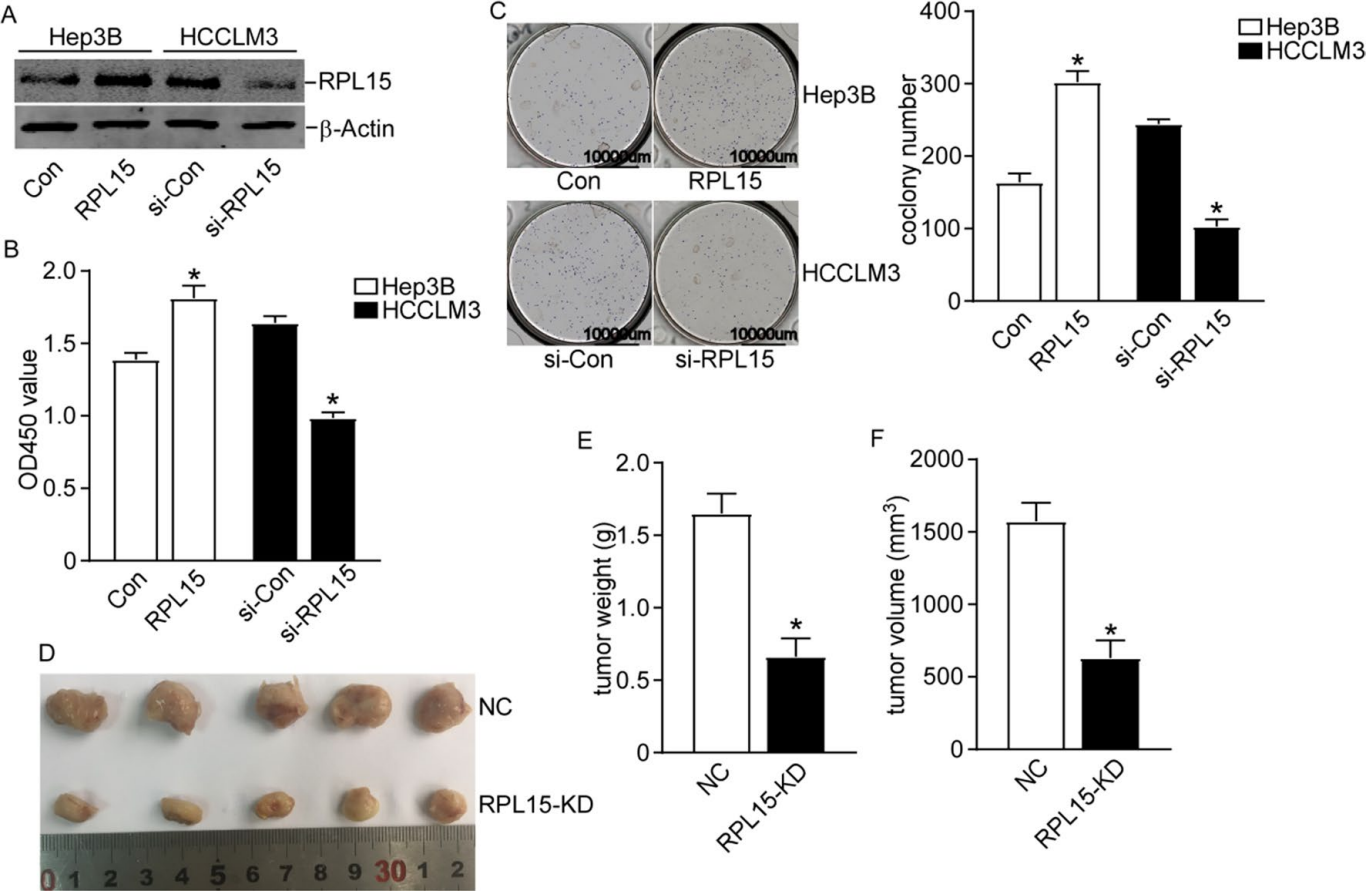
RPL15 downregulation could inhibit HCC cells proliferation in vivo and in vitro. **A** The efficiency of RPL15 overexpression in Hep3B and knockdown in HCCLM3 cells was confirmed by western blot. **B** CCK-8 assays showed that Hep3B cells with RPL15 overexpression displayed more active proliferative capacity while the RPL15-silenced HCCLM3 cells had weaker proliferative ability. **C** Results of colony formation assays showed that RPL15 knockdown significantly reduced while RPL15 overexpression increased the number of colonies. **D** Representative tumor image of xenograft mice. **E, F** Tumor volumes and weights of HCC cell xenograft model in RPL15-knockdown group were significantly reduced. Data are presented as the mean ± SD, n = 3; *P < 0.05, compared to the control group. NC means negative control; RPL15-KD, RPL15 stable knockdown

As the association between RPL15 and HCC prognosis had been verified, we next investigated the functional roles of RPL15 in HCC cell growth. HCCLM3 cells exhibited higher expression level of RPL15, and the expression in Hep3B was lower, the RPL15 was overexpressed in Hep3B cells and knocked down in HCCLM3 cells (Fig. 3A). Then, CCK-8 assay and colony formation assay were performed in Hep3B and HCCLM3 cells. Hep3B cells with RPL15 overexpression showed more active proliferative capacity while the RPL15-silenced HCCLM3 cells had weaker proliferative ability (Fig. 3B). Similarly, the same results were obtained from colony assay. RPL15 overexpression significantly increased while RPL15 knockdown reduced the number of colonies (Fig. 3C). These data showed that RPL15 downregulation could inhibit HCC cell proliferation. Thereafter, we further investigated the effects of RPL15 on HCCLM3 cells growth in vivo. Tumor volumes and weights in RPL15-knockdown group were significantly reduced compared to the NC group (Fig. 3D, E, F). These results demonstrated that RPL15 downregulation could inhibit HCC cell growth.

### RPL15 knockdown blocked cell cycle progression in HCC cells

**Fig. 4:**
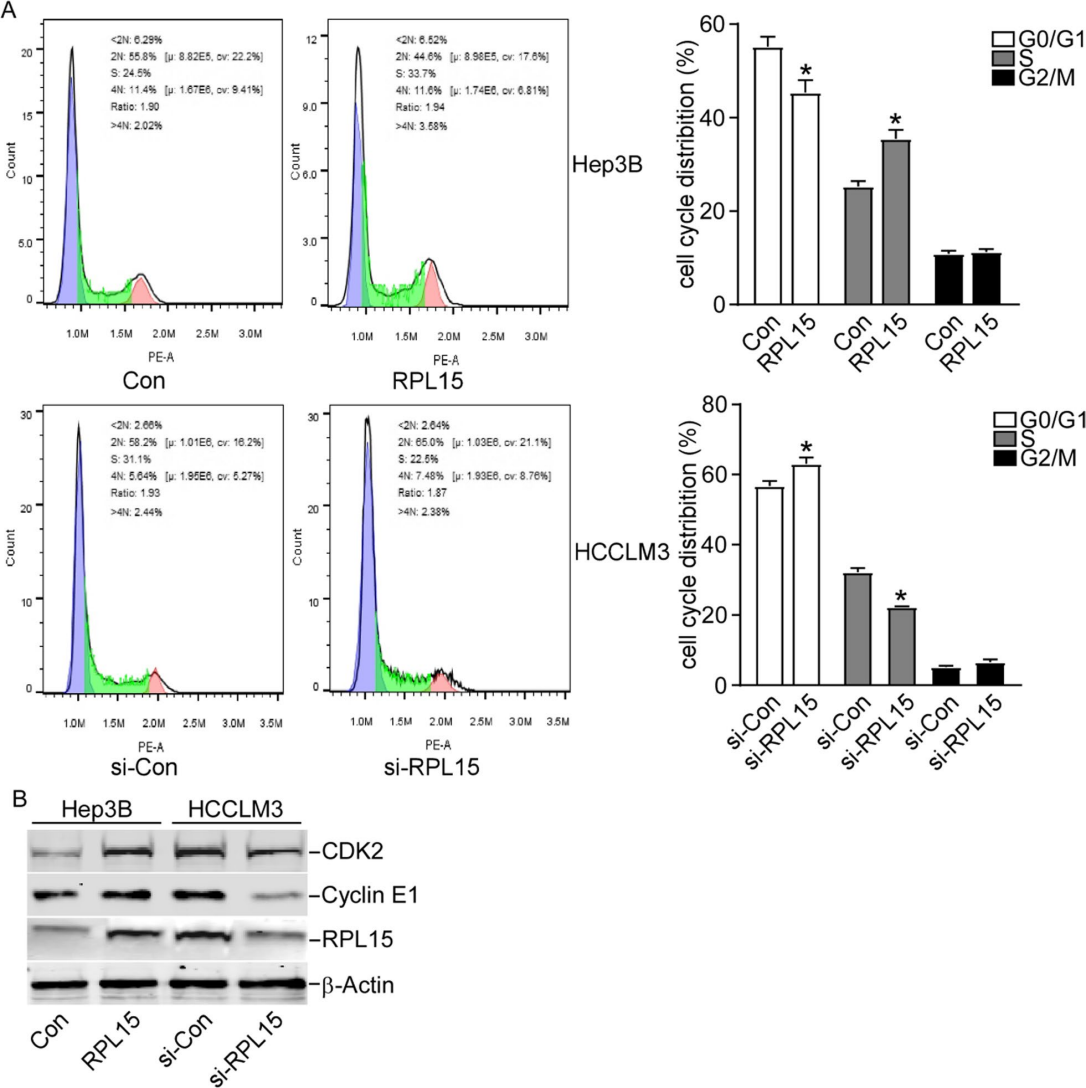
RPL15 knockdown blocked cell cycle progression in HCC cells. **A** RPL15 knockdown in HCCLM3 cells induced the cell cycle arrest in G1 phase while RPL15 overexpression in Hep3B cells induced the converse effects. **B** Western blot analysis revealed that RPL15 silencing decreased CDK2 and Cyclin E1 expressions, while RPL15 overexpression promoted their expressions. Data are presented as the mean ± SD, n = 3; *P < 0.05, compared to the control group

To explore the underlying mechanism of the regulatory effect of RPL15 on HCC cell proliferation, the cell cycle distribution of HCC cells was measured. RPL15 overexpression in Hep3B cells promoted the cell cycle from G1 to S phase, RPL15 knockdown in HCCLM3 cells induced the cell cycle arrest in G1 phase (Fig. 4A). Furthermore, the levels of cell cycle-related genes were also detected by Western blotting. RPL15 overexpression increased CDK2 and Cyclin E expressions in Hep3B cells, while RPL15 silencing had the opposite effect in HCCLM3 cells (Fig. 4B).

### RPL15 knockdown enhanced HCC cells apoptosis

**Fig. 5:**
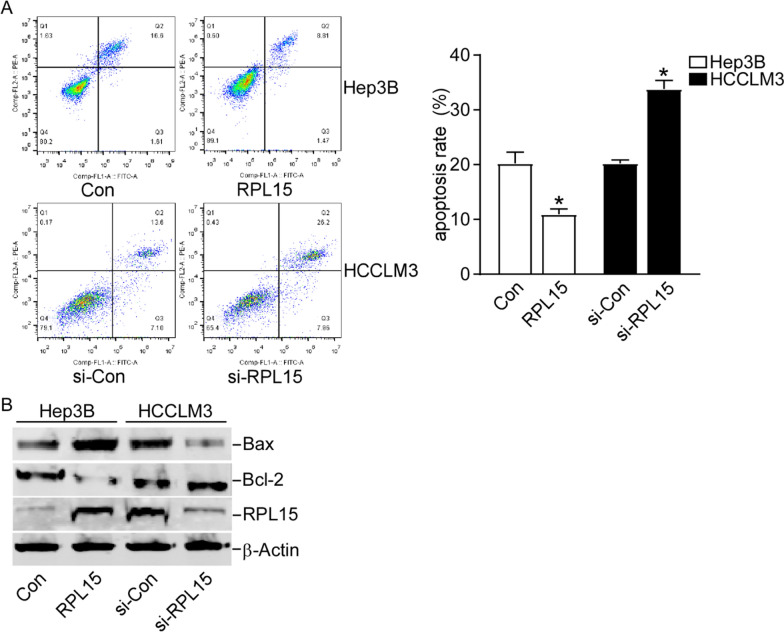
RPL15 knockdown enhanced HCC cells apoptosis. **A** RPL15 knockdown promoted HCCLM3 cell apoptosis, and RPL15 overexpression prevented Hep3B cell apoptosis**. B** Western blot analysis revealed that RPL15 knockdown upregulated the expression of Bax and downregulated the expressions of Bcl-2, and RPL15 overexpression induced the converse effects. Data are presented as the mean ± SD, n = 3; *P < 0.05, compared to the control group

The effect of RPL15 on HCC cell apoptosis was investigated by flow cytometry. RPL15 overexpression prevented the Hep3B cells apoptosis, and RPL15 knockdown promoted the HCCLM3 cells apoptosis (Fig. 5A). Then, the levels of several apoptosis-related genes were measured to further explore the apoptosis mechanism. Western blot analysis revealed that RPL15 overexpression decreased the expression of Bax and increased the expressions of Bcl-2, while RPL15 knockdown had inverse effect on Bax and Bcl-2 (Fig. 5B). In general, the findings demonstrated that RPL15 could suppress apoptosis in HCC cells.

### RPL15 silencing decreased migration and invasion abilities in HCC cells

**Fig. 6:**
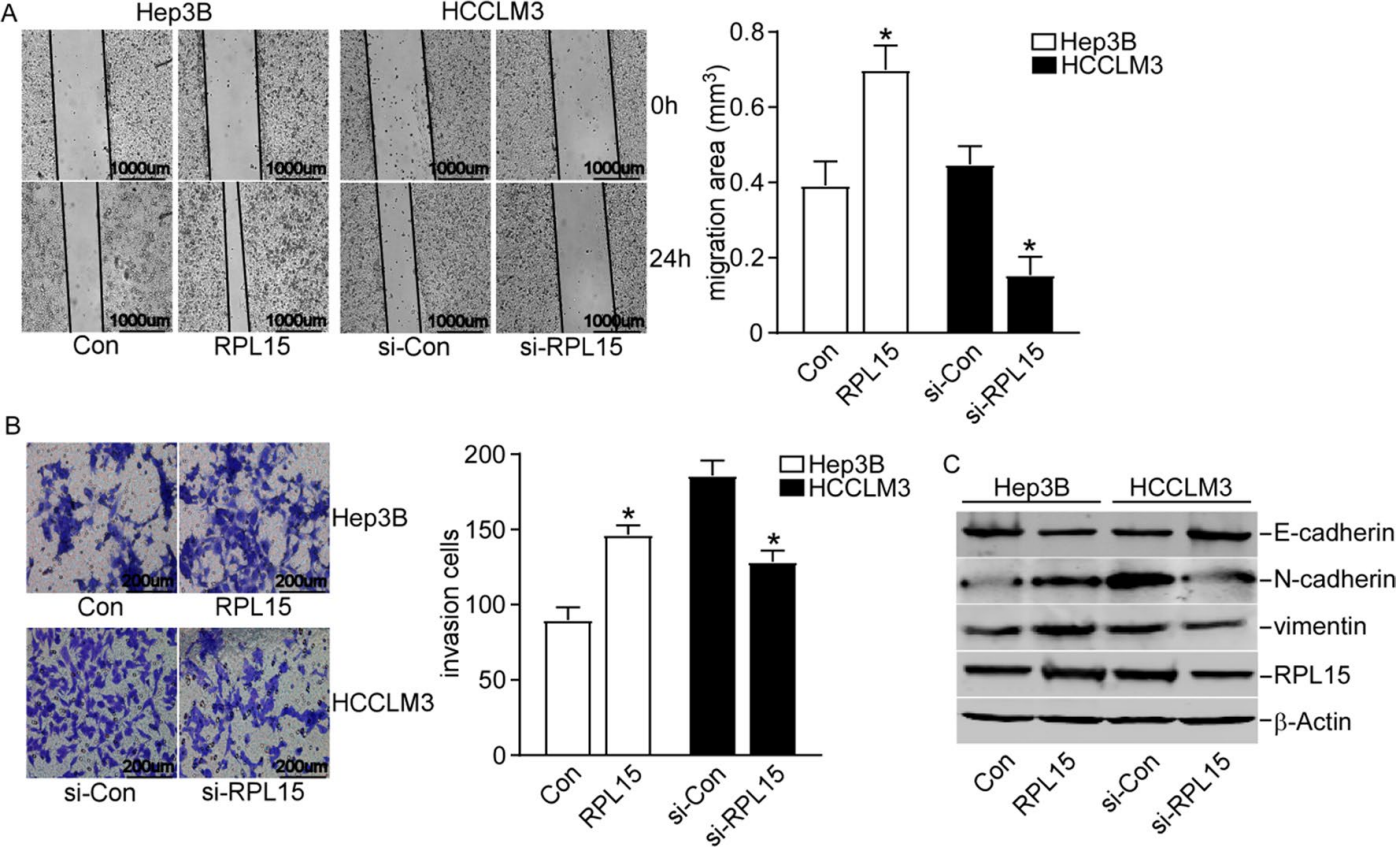
RPL15 silencing inhibited HCC cells migration and invasion. **A** Wound-healing assay indicated that RPL15 silencing reduced HCCLM3 cell migration while RPL15 overexpression enhanced Hep3B cell migration. **B** Transwell assay revealed that RPL15 knockdown decreased the invasion capability of HCCLM3 cells while overexpression of RPL15 in Hep3B cells demonstrated the opposite effect. **C** The expressions of EMT-related genes were detected after RPL15 was silenced or overexpressed in HCC cells via western blot. Data are presented as the mean ± SD, n = 3; *P < 0.05, compared to the control group

To further evaluate the function of RPL15 in HCC cells on migration and invasion, the wound-healing assay and transwell assay were employed. RPL15 overexpression enhanced the Hep3B cells migration, while RPL15 silencing reduced the HCCLM3 cells migration (Fig. 6A). Consistently, the transwell assay also indicated that overexpression of RPL15 increased the invasion capability, while RPL15 knockdown demonstrated the opposite effect (Fig. 6B). Given that tumor cell invasion and migration were usually associated with epithelial–mesenchymal transition (EMT), we further assessed the expression of EMT-associated markers by western blot analysis. RPL15 overexpression reduced E-cadherin expression, but increased the expression of N-cadherin and vimentin in Hep3B cells (Fig. 6C). Remarkably, RPL15 knockdown caused the opposite results in HCCLM3 cells (Fig. 6C). Such findings suggested that RPL15 promoted HCC cell invasion and migration by regulation of EMT.

### RPL15 involved in RPs-MDM2-p53 signaling pathway

**Fig. 7:**
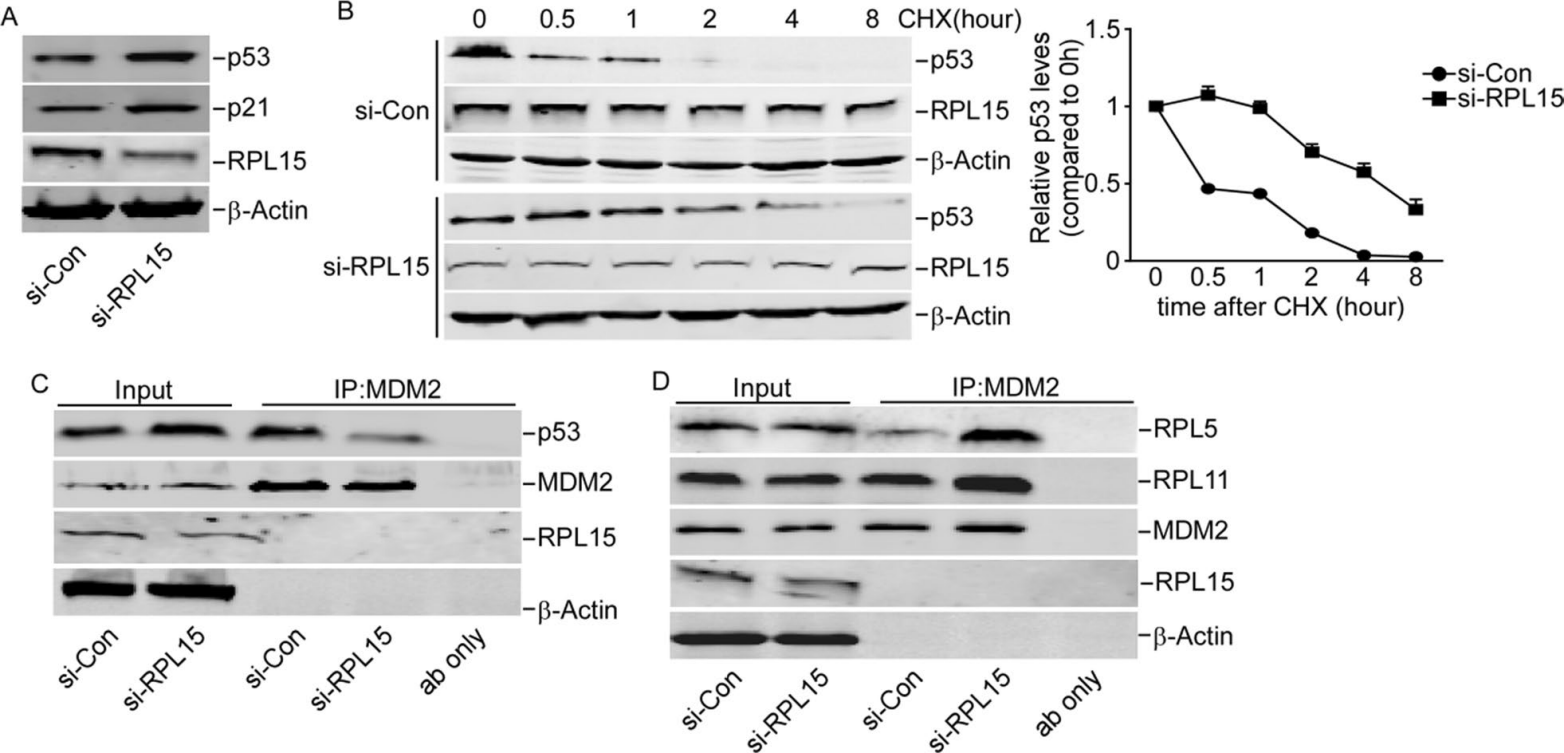
RPL15 involved in RPs-MDM2-p53 signaling pathway. **A** Western blot analysis revealed that the levels of p21 and p53 were significantly increased in HCCLM3 cells with RPL15 knockdown**. B** Western blotting from 0–8 h after cycloheximide (CHX) treatment to stop protein synthesis showed more stability of p53 in HCCLM3 cells with RPL15 knockdown. **C** RPL15 silencing suppressed the interaction of MDM2 with p53, the binding was determined by immunoprecipitation. **D** Effect of RPL15 on the binding of MDM2 with RPL11 and RPL5 was determined by immunoprecipitation, RPL15 silencing suppressed the interaction

As one of the primary “gatekeepers” of the cell, the p53 tumor suppressor plays a major role in sensing and responding to a variety of stressors to maintain cellular homeostasis. Recent studies have shown that perturbations in ribosomal biogenesis tightly associated with cellular activities, can affect p53 through ribosomal protein (RP)-mediated MDM2 E3 ligase activity [19, 20]. Was whether p53 which would accelerate cell apoptosis involved in the biological function of RPL15 in regulation of HCC cell progression, p53 and p21 targeted by p53 was further explored. The levels of p53 and p21 were significantly increased in HCCLM3 cells with knockdown of RPL15 (Fig. 7A).Then, the RPL15 didn’t affect the transcriptional activities of p53 (Additional file 1: Figures SA, SB and SC). As MDM2 is an important ubiquitin E3 ligase that can bind to p53 to promote its degradation, the p53 degradation and the interaction between MDM2 were explored in HCCLM3 cells by RPL15 knockdown. RPL15 knockdown inhibited the p53 degradation while HCCLM3 cells were treated with cycloheximide (CHX) inhibiting protein synthesis (Fig. 7B). And RPL15 knockdown also inhibited the binding of MDM2 to p53 (Fig. 7C). These results indicated that RPL15 silencing suppressed MDM2-mediated degradation of p53. The ability of ribosomal proteins L5, L11, L23, L26, or S7 to bind MDM2 and inhibit its ubiquitin ligase activity has been suggested as a critical step in p53 activation [21–23]. Then, the binding of RPL5 and RPL11 with MDM2 in HCCLM3 cells of RPL15 knockdown was verified. Results indicated that the binding of MDM2 to RPL5 or RPL11 in HCCLM3 cells was prominently elevated when RPL15 silenced (Fig. 7D). These results indicated that the knowdown of RPL15 enhanced the interaction between RPL5/11 and MDM2, inhibited MDM2-mediated p53 degradation that leads to p53 stabilization and activation.

### RPL15 mediated HCC progression via the RPs-MDM2-p53 signaling pathway

**Fig. 8:**
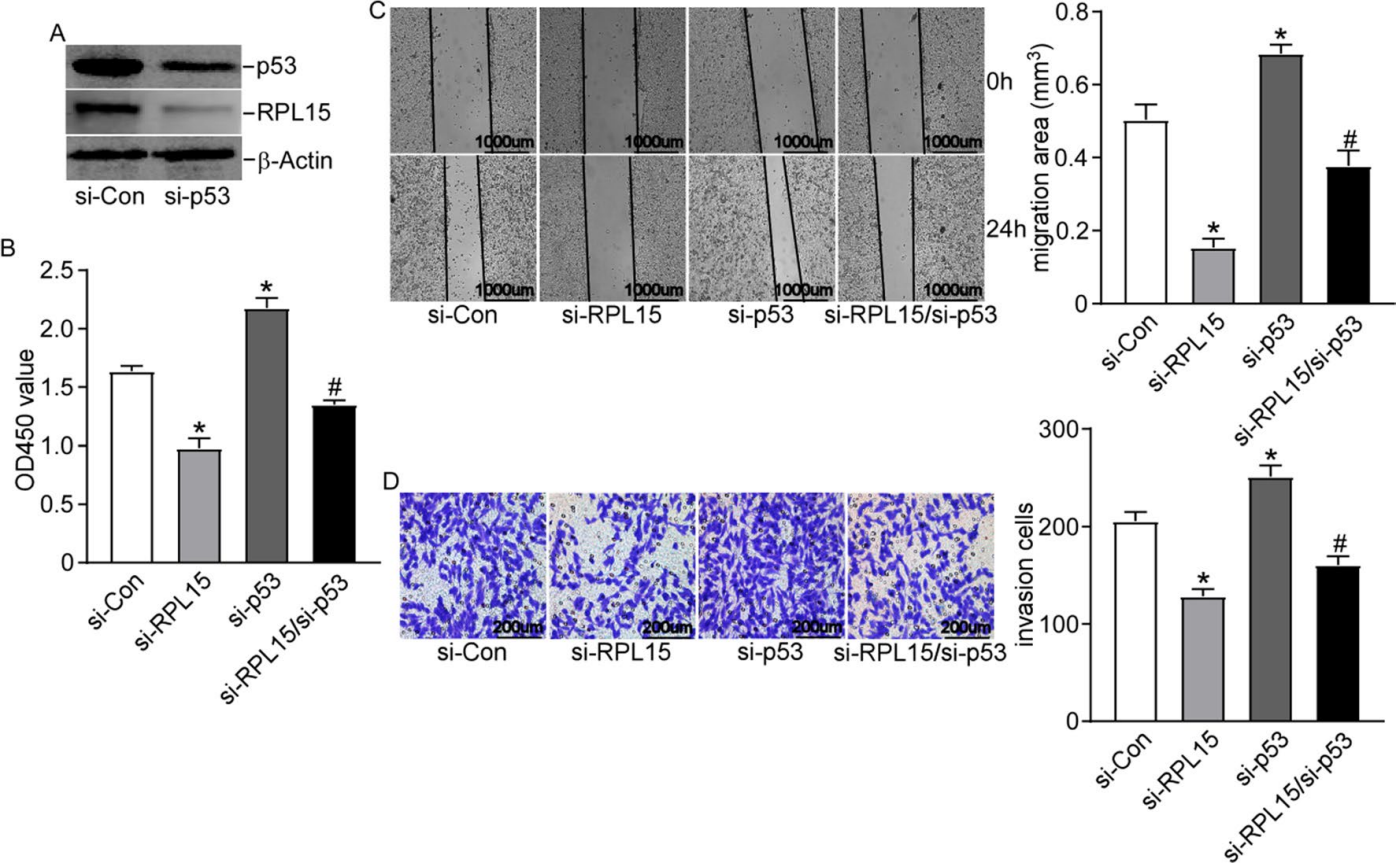
RPL15 mediated HCC progression via the RPs-MDM2-p53 signaling pathway. **A** The efficiency of p53 knockdown in HCCLM3 cells was confirmed by western blot. **B** CCK-8 assays showed that si-p53 reversed the inhibitory function of proliferation in HCCLM3 cell with RPL15 knockdown. **C** Wound-healing assay indicated that si-p53 reversed the inhibitory function of migration in HCCLM3 cell with RPL15 knockdown. **D** Transwell assay revealed that si-p53 reversed the inhibitory function of invasion in HCCLM3 cell with RPL15 knockdown. Data are presented as the mean ± SD, n = 3; *P < 0.05, compared to the control group; #P < 0.05, compared to the si-RPL15 group

As RPL15 could potentially regulate the degradation of p53 mediated by RP5/11-MDM2, we then examined the function of RPL15-MDM2-p53 pathway in modulating HCCLM3 cell proliferation, invasion, and migration. HCCLM3 cells were transfected with Si-RPL15 and/or si-p53 (Fig. 8A), and then underwent CCK8, scratch wound healing, and Transwell invasion assays. Si-RPL15 inhibited the proliferation, invasion, and migration of HCCLM3 cells, which were greatly reversed by co-transfection of si-p53 (Fig. 8B–D). These results indicated that RPL15 might promote HCC progression partly via suppressing the RPs-MDM2-p53 signaling pathway.

## Discussion

HCC is one of the most common malignant tumors of the digestive system, which seriously threatens human health and survival. Moreover, the surgical treatment is limited and drug treatment also has obvious side effects and drug resistance [24]. The occurrence and development of HCC is a complex process involving multiple factors and multiple steps. The most important biological characteristics of cancer cells are the unrestrained rapid proliferation due to abnormal signal transduction of cell proliferation, uncontrolled cell growth cycle, genomic instability, and epigenetic changes. At the same time, in order to meet the need for rapid proliferation, tumor cells also show a different ability to ribosome biogenesis from normal tissue cells [25]. With the advancement of precision medicine and individualized medicine, gene-targeted therapy predicts and intervenes in advance during the development of HCC via seeking more direct and effective anti-HCC drug molecular targets. Gene-targeted therapy overcomes the limitations and side effects of traditional treatment and has become an important direction in HCC treatment.

In recent years, a large number of studies have shown that RPL11, RPL5 and 5S rRNA form a protein-nucleic acid complex that binds to and inhibits the E3 ubiquitin ligase activity of the oncogene MDM2, which could ubiquitinate the tumor suppressor gene p53 and regulate the level of intracellular p53 protein [26–28]. The mutations of RPL11 and RPL5, the abnormally high expression of the oncogene MDM2, and the abnormal function of the tumor suppressor gene p53 could lead to the occurrence and development of a variety of tumors [29]. In a variety of tumor cells, knocking down the ribosomal subunit protein can cause the cells to produce a stress response, that is ribosome stress. Under ribosomal stress condition, RPL11 and RPL5 are released from the nucleolus into the nuclear matrix, and they can form a complex with 5S rRNA and MDM2, thereby inhibiting the ubiquitination of p53 caused by MDM2, leading to an increase in p53 level and activating the cell cycle check point, causing cell cycle block [30, 31]. Several nucleolar proteins stabilize p53 by interfering RPs-MDM2–p53 interaction upon cellular stress, while other mechanisms by which nucleolar proteins activate p53 remain to be determined. RPS26 induced p53 stabilization and activation via a RPL11-dependent mechanism, resulting in p53-dependent cell growth inhibition. Moreover, RPS26 also has the ability to interact with MDM2 and inhibits MDM2-mediated p53 ubiquitination that leads to p53 stabilization upon overexpression [23]. NAT10 played a critical role in p53 activation via acetylating p53 and counteracting MDM2 action [32]. SNORA18L5 increased ribosome biogenesis, and alters localization of RPL5 and RPL11, allowing for increased MDM2-mediated proteolysis of p53 and cell cycle arrest [21]. Through the ribosomal stress response pathway, cells can respond to various stress stimuli, thereby maintaining cell homeostasis and genome stability. Abnormalities in this response pathway can also lead to a variety of diseases. In summary, the abnormality of ribosomal biogenesis is closely related to the occurrence and development of many human diseases, especially tumors. Targeting the pathway of ribosomal biogenesis can provide a new approach for tumor treatment.

In current study, the oncogenicity of RPL15 and its mechanism in HCC were investigated. The results of the present study showed that RPL15 was overexpressed in HCC tissues, which was associated with malignant clinicopathological characteristics of HCC patients. Furthermore, overexpression of RPL15 predicted a worse prognosis and shorter survival in HCC patients. The results of the functional studies revealed that RPL15 had great tumorigenicity, which could promote cell growth, migration, and invasion while inhibit cell apoptosis in HCC cell lines. These effects were effectively inhibited by RPL15 knockdown. Cell proliferation is usually related to apoptosis and/or cell cycle. Results of our study showed that RPL15 knockdown could induce the cell cycle arrest in the G1 phase in HCCLM3 cells. Furthermore, the expression of cell cycle-related genes (CDK1 and Cyclin B1) in HCC cells were also downregulated by RPL15 knockdown. In general, we concluded that RPL15 could reduce cell cycle arrest in the G1 phase. RPL15 knockdown was also found to decrease the expression of anti-apoptotic proteins Bcl-2 and increase the expression of tumor suppressor gene Bax. Hence, we investigated whether the changes in cell motility and metastasis were associated to the EMT process by RPL15. RPL15 knockdown in HCCLM3 cells increased E-cadherin expression, but reduced the expression of N-cadherin and vimentin, which confirmed our assumption. We also found that RPL15 was capable of modulating the RPs-MDM2-p53 pathway to regulate HCC cell progression. The knockdown of RPL15 inhibited MDM2-mediated p53 ubiquitination that leads to p53 stabilization and activation via a RPL5/11-dependent mechanism, resulting in p53-dependent cell proliferation, invasion and migration. The knockdown of RPL15 enhanced the interaction between RPL5/11 and MDM2. One reason was that disruption of small subunits of ribosomes leads to an enhanced translation of RPL5/11 through a 5′-top-mediated mechanism [33]. The other is that disruption of ribosomal subunit enhanced the nuclear retention of RPL5/11, thus increasing its interaction with MDM2 [34].

In conclusion, RPL15 was overexpressed in HCC and correlated with poor prognosis in HCC. We proposed a potential carcinogenic role of RPL15 in HCC cells. Indeed, RPL15 was involved in cell proliferation, apoptosis, migration and invasion during HCC carcinogenesis and development via regulation of p53 signaling. Overall, our findings indicated RPL15 as a novel diagnostic marker and therapeutic target in HCC.

## Supplementary Information

**Additional file 1: Figure SA and SB.** The efficiency of RPL15 overexpression in Hep3B and knockdown in HCCLM3 cells was confirmed by qRT-PCR and western blot.** Figure SC.** The levels of p53 in HCCLM3 cells with RPL15 overexpression and knockdown was revealed by qRT-PCR**.**

## Data Availability

The datasets used and/or analyzed during the current study are available from the corresponding author upon reasonable request.
