# Supplementary material for: RPL15 promotes hepatocellular carcinoma progression via regulation of RPs-MDM2-p53 signaling pathway
Source: Cancer Cell Int. 2022 Apr 11;22:150. doi: 10.1186/s12935-022-02555-5 (PMC9003963; doi:10.1186/s12935-022-02555-5)
Supplement: Supplementary file 1 — Additional file 1: Figure SA and SB. The efficiency of RPL15 overexpression in Hep3B and knockdown in HCCLM3 cells was confirmed by qRT-PCR and western blot. Figure SC. The levels of p53 in HCCLM3 cells with RPL15 overexpression and knockdown was revealed by qRT-PCR. [file 12935_2022_2555_MOESM1_ESM.docx]

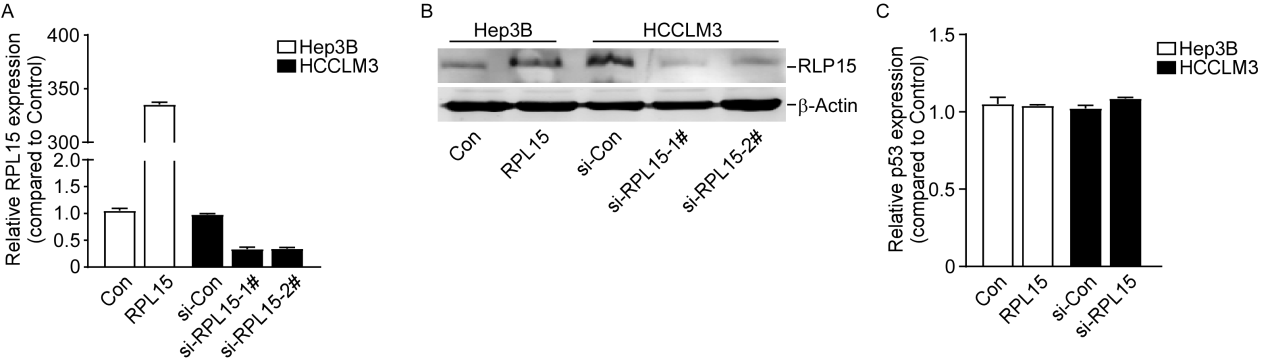


**Additional file 1: Figure. SA and SB** The efficiency of RPL15 overexpression in Hep3B and knockdown in HCCLM3 cells was confirmed by qRT-PCR and western blot. S**C** The levels of p53 in HCCLM3 cells with RPL15 overexpression and knockdown was revealed by qRT-PCR**.**
